# Habit-Forming Drugs in Proprietary Medicines. (Ibid)

**Published:** 1916-02

**Authors:** 


**Habit-Forming Drugs in Proprietary Medicines. (ibid).** The same 1108 proprietaries were considered, the statements made on the labels being accepted as true, doubtless because the risk of untrue statements is obviously fatal to the continuance of the business. None contained cocaine in "any quantity." Whether this expression is to be taken literally or in its colloquial meaning is not stated. 3 contained cannabis indica, 2 being corn remedies and one a cough mixture which would be toxic by virtue of its other constituents before the effect of the cannabis indica would become serious. None contained chloral. 28 external remedies contained opium or an alkaloid of opium. In 20, the content exceeded and in 5 it was less than the standard of 2 grains of opium or ¼ grain of morphine per ounce. 42 cough and diarrhoea mixtures and the like contained opiates but only in excess of the preceding standard and only one was in excess of the average opium content of 5 N. F. formulas for diarrhoea. 4 preparations were soothing syrups or powders for children. This study was made of preparations put on the market before the enactment of the Harrison law.

